# Effects of polygenes, parent–child relationship and frustration on junior high school students' aggressive behaviors

**DOI:** 10.1002/pchj.717

**Published:** 2023-12-27

**Authors:** Minghao Zhang, Zhenli Jiang, Kedi Zhao, Yaohua Zhang, Min Xu, Xiaohui Xu

**Affiliations:** ^1^ School of Educational Science Ludong University Yantai China; ^2^ Collaborative Innovation Center for the Mental Health of Youth from the Era of Conversion of New and Old Kinetic Energy along the Yellow River Basin, Ludong University Yantai China; ^3^ College of Safety and Environmental Engineering Shandong University of Science and Technology Qingdao China; ^4^ Factor‐Inwentash Faculty of Social Work University of Toronto Toronto Ontario Canada

**Keywords:** aggressive behaviors, frustration, junior high school students, parent–child relationship, polygenic risk score

## Abstract

The effects of the interaction between polygenes and the parent**–**child relationship on junior high school students' aggressive behaviors were explored through the frameworks of gene‐endophenotype‐behavior and neurophysiological basis. A total of 892 junior high school students participated in this study. They were asked to complete self‐reported questionnaires, and saliva samples were collected. Results showed that 5‐HTTLPR, MAOA‐uVNTR, COMT (rs4680), and *Taq*1 (rs1800497) of the DRD2 gene affected students' aggressive behaviors in an accumulative way. The polygenic risk score explained 3.4% of boys' aggression and 1.1% of girls' aggression. The interactions between polygenic risk score and parent**–**child conflict significantly affected the aggressive behaviors of male students, but did not show any significant effect on those of female students. The interactional effect of polygenic risk score and parent**–**child conflict on junior high school students' aggressive behaviors was completely mediated by frustration. However, the interaction effect of polygenic risk score and parent**–**child affinity on aggression was not affected by frustration. This study helps us better understand junior high school students' aggressive behaviors and promotes the prevention and correction of adolescents' problem behaviors.

## INTRODUCTION

Adolescents' aggressive behaviors are often related to problematic behaviors later in life, such as alcoholism, drug abuse, violent crimes, suicide, and depression (Aliev et al., 2014). Previous twin and adoptive studies, as well as a meta‐analysis, found that aggressive behaviors are influenced by concurrent heredity and environment, and 50% of variances are attributed to genes (Tuvblad & Baker, 2011); yet, most of these studies investigated aggressive behaviors from the monogenic × environmental perspective (e.g., Tielbeek et al., 2012). However, the monogenic perspective can only partially explain adolescents' aggressive behaviors, and results from these monogenic studies are often inconsistent (Fernàndez‐Castillo & Cormand, 2016). With the development of the genome‐wide association study, the joint effects patterns of polygenic loci on behavior have gradually become more prevalent (Armstrong‐Carter et al., 2021). The present study focused on 5‐hydroxytryptamine and dopamine genes that relate to aggressive behaviors, and examined the influences of polygenes and parent**–**child relationships on adolescents' aggressive behaviors.

In addition to the influences of gene–environment interplay, the general aggression model (GAM) put forward by Anderson and Bushman (2002) suggests that individual characteristics, such as cognition and personality traits, can also affect adolescents' antisocial behaviors. This theoretical framework accentuated the mediating roles of cognitive and emotional functioning, as well as neurobiological deficits, in children’s antisocial behaviors and their early adversity (van Goozen et al., 2007). The theoretical framework of “gene‐endophenotype‐behavior” also highlights that individual characteristics related to genetic and internal stability, such as neurobiological characteristics, emotions, and temperament, may impact the relationship between genes and behaviors (e.g., Chan et al., 2008). More specifically, genes provide the biological foundation for aggressive behaviors, while the external environment provides the environmental foundation. Individual factors such as cognition and personality traits may be intermediate mechanisms of the effects of heredity and environment on aggressive behaviors. Therefore, this study aimed to examine the role of frustration in polygenes × environment and aggressive behaviors in order to provide more evidence on the causes of aggressive behaviors.

### Polygenes and aggressive behaviors

In recent years, genetic research on aggressive behaviors has focused mainly on monoamine neurotransmitter and genes in the serotonin (5‐HT) and dopamine system (Åslund et al., 2013; Byrd & Manuck, 2014; Caspi et al., 2002; Kim‐Cohen et al., 2006). For example, 5‐HTTLPR is a polymorphism in the promoter sequence of the serotonin transporter gene. Owing to the insertion or deletion of a 44‐bp promoter region, 5‐HTTLPR forms long (L) and short (S) alleles. A previous study showed that among those who experienced child abuse, participants that carried 5‐HTTLPR S/S and S/L were more likely to exhibit violent behaviors than those who carried L/L (Reif et al., 2007).

Even though a single gene can affect aggressive behaviors, its associations with aggressive behaviors are limited and require a large sample size to get statistically significant results. Previous studies found that the explanation by polymorphism of monogenic loci accoutned for only 1%, while genome‐wide association studies found that 13 SNPs accounted for 5.2% of antisocial behavior (single nucleotide polymorphisms) (Harden & Koellinger, 2020; Tielbeek et al., 2017). Nikolova et al. (2011) conducted a meta‐analysis that included 103 studies and found that the additive effects of five alleles on dopamine signaling explained 10.9% of the variability in reward‐related reactivity. In addition, Langevin et al. (2019) found that for serotonin, the polygenic effects of 11 genes predicted 9.6% to 15.2% of adolescents' violence and crimes. Hence, polygenic effects can better predict variability in behavior. For instance, DRD2 (dopamine D2 receptor) and DRD4 (dopamine D4 receptor) found in dopamine did not have significant independent effects, whereas the interaction between DRD4 7R and A1 in DRD2 predicted an increase in aggressive behaviors (e.g., Beaver et al., 2007).

Therefore, complex behaviors are not the outcome of one specific gene or the variability of one genetic locus; they may be the outcome of two or more combinations of polygenetic variabilities (Raffington et al., 2020; Uchiyama et al., 2021). In China, there is still a dearth of hereditary studies that investigate aggressive behaviors from a polygenic perspective, and it is unclear whether serotonin and dopamine affect aggression in an accumulative or interactive manner. Hence, the first goal of this study was to explore how the polymorphism of 5‐HTTLPR, MAOA‐uVNTR (a functional variable number of tandem repeats‐polymorphism in the promoter region of the MAOA gene), COMT (catechol‐O‐methyltransferase) (rs4680), and *Taq*1 (rs1800497) of the DRD2 gene affected junior high school students' aggressive behaviors from a hereditary perspective.

### Parent–child relationship and aggressive behaviors

The parent–child relationship affects the development of adolescents in two ways. On the one hand, conflicts between parents and children have negative effects on children's emotions and behaviors. For example, high levels of parent–child conflict and rejection contribute to adolescents' external problems (e.g., aggression and antisocial behavior) and internal problems (e.g., depression and anxiety) (Rohner & Britner, 2002; Sentse & Laird, 2010). On the other hand, high levels of parent–child affinity prevent adolescents from having social behavioral problems. For example, Zhao et al. (2013) found in 424 rural children from 10 to 17 years old that for left‐behind children whose parents go out to work, the prediction of peer rejection on aggression against child and disciplinary violations is affected by the level of parent‐child affinity. Specifically, with a higher level of parent–child affinity, the impact of peer rejection on child aggression and disciplinary violations is reduced.

In conclusion, the parent–child relationship is an important factor affecting aggressive behavior.

### Interactions between polygene and environment

There are three hypotheses to explain the effects of the interactions between genes and environment on behaviors: the diathesis‐stress model, the differential susceptibility model, and the vantage sensitivity model (Jolicoeur‐Martineau et al., 2017).

The diathesis‐stress model posits that some alleles are risky and only sensitive to a negative environment. When in an adverse environment, individuals who carry risk alleles are more likely to display problematic behaviors, such as aggression, rule violation, violence, and impulsivity (De Laet et al., 2016). When individuals are in a positive environment, their development is not affected by the effects of the variabilities of alleles. The differential susceptibility model proposes that some alleles will increase the onset of aggressive behaviors in an adverse situation but lower the risk of individuals exhibiting aggressive behaviors in a positive condition (Belsky et al., 2009). This model describes these kinds of alleles as plasticity alleles. In other words, plasticity alleles can impact individuals either positively or negatively (Pluess & Belsky, 2013). Finally, the vantage sensitivity model depicts the variabilities of specific genes in positive environmental conditions. Individuals with hereditary advantages may have more positive reactions to a supportive environment, whereas those without these specific genes may be less interactive in a positive environment (Cleveland et al., 2015). However, compared with their negative counterparts, positive environments and behaviors have still not been extensively investigated in hereditary research. Because the vantage sensitivity model is recent, there is still a dearth of empirical studies investigating this model (e.g., Pluess, 2017; Pluess & Belsky, 2013).

Hence, the second goal of this study was to explore the effects of polygenes × parent**–**child relationships on aggressive behaviors, and to further examine whether the interactions between polygenes and the parent**–**child relationship can be explained by any of the models mentioned above.

### The effects of polygenes and parent–child relationship on junior high school students' aggressive behaviors: The role of frustration

The general aggression model (Anderson & Bushman, 2002) and the “gene‐endophenotype‐behavior” framework (Chan et al., 2008) both note that individual characteristics with genetic and internal stability affect both genes and behaviors. Individual variability in temperaments is regarded as highly heritable (from 35% to 57%; Bouchard & Thomas, 2004). Frustration, as a temperament, may also have high heredity, but there are no empirical studies to explain its genetic effects. Pawliczek et al. (2013) found that frustration relates to the prefrontal cortex, amygdala, and striatum. Because the structure and the function of these areas are affected by genetic polymorphisms of serotonin and dopamine, frustration may also be affected by these genes. For instance, Davies and Cicchetti (2014) found that the interactions of a mother's parenting style and 5‐HTTLPR polymorphism can predict infants' emotional development. Because frustration affects aggression, and frustration and aggression are similarly heritable, frustration might thus be the endophenotype of the effects of genes on aggressive behaviors. The effects of the interactions between genes and environment on aggressive behaviors might also be achieved through the endophenotype, frustration. Hence, the third goal of this study was to explore the mediating role of frustration in the effects of the interactions between polygenes and parent**–**child relationships on junior high school students' aggressive behaviors.

## METHODS

### Participants

This study used the cluster sampling method. The sample comprised 1072 students from two junior high schools in a coastal city in China. Specifically, 24 classes from grades six through eight were selected, made up of eight classes in each grade. Each class had between 35 and 50 students. All parents provided written informed consent. A total of 72 questionnaires were invalid, and 108 saliva samples were missing. There were 892 students with both valid questionnaires and genetic data. Participants were all ethnic Han Chinese between the ages of 10 and 17 (436 males and 456 females; *M*_age_ = 13.43 years old, *SD* = 1.00).

### Procedure

Ethics approval from the Ethics Science Committee of the School of Educational Science at Ludong University was obtained to conduct the study. Consent forms were distributed to parents, and students from grades six to eight who obtained permission from both the school and their parents participated in the study. Prior to the study, master's students in the psychology program were recruited as research assistants and were further trained so that they became familiar with the study materials and procedures. Specifically, two master's students distributed questionnaires to students in the classroom and collected them after completion. Students' exfoliative oral cells were also collected so that their genetic data could be analyzed. To do this, participants were first asked to rinse their mouths with saline, and then to insert the oral swab head into the oral cavity. Students were told that the oral swab should fully contact the inside cheeks (both left and right) and be rubbed up and down thoroughly against both cheeks 30 times. After completing this procedure, the oral swab was stored in the sampling tube. Research assistants then collected participants' sampling tubes and ensured that the oral swabs were properly stored in the sampling tubes.

The extraction and genotyping of DNA was assisted by Beijing TsingKe Biological Technology Company. The VNTR polymorphism of 5‐HTTLPR (S & L alleles) and MAOA‐uVNTR (H & L alleles) and the SNP polymorphism of COMT (rs4680, Val & Met alleles) and *Taq* 1 of DRD2 (rs1800497, A1 & A2 alleles) were analyzed.

### Aggressive behaviors

The Chinese version of the aggression questionnaire (Buss & Perry, 1992) was used to measure junior high school students' self‐reported aggressive behaviors. This questionnaire uses a 5‐point Likert scale to document participants' scores; higher scores represent more frequent aggressive behaviors. The Cronbach *α* of this questionnaire was .88.

### Parent–child relationships

The Network of Relationships Inventory (Furman & Buhrmester, 1985) was used to measure parent**–**child conflict (father**–**child conflict and mother**–**child conflict) and parent**–**child affinity (father**–**child affinity and mother**–**child affinity). A 5‐point Likert scale was again used to calculate participants' scores. The higher the score, the higher the chance that the participant has parent**–**child affinity and parent**–**child conflict. The Cronbach ɑs of parent**–**child affinity and parent**–**child conflict were .92 and .89, respectively.

### Frustration

The Early Adolescent Temperament Questionnaire (EATQ‐R; Ellis & Rothbart, 2001) was used to measure adolescents' frustration. Participants were asked to choose an answer on a 5‐point Likert scale. A high score means that the participant is more inclined to be frustrated. The Cronbach ɑ of this questionnaire was .70.

### Data analyses and interpretation

First, SHESIS (Shi & He, 2005) was used to examine whether the allele distributions of 5‐HTTLPR, MAOA‐uVNTR, COMT (rs4680), and *Taq*1 (rs1800497) of the DRD2 gene comply with the Hardy–Weinberg equilibrium. A multilevel regression model was used to examine the effects of interactions between polygenes and parent**–**child affinity/conflict on junior high school students' aggressive behaviors using SPSS22.0 (IBM Corp, 2013). CPGS scores and parent–child relationships were entered first, and then the interaction between them. Mplus 7.4 (Muthén & Muthén, 1998‐2017) was then used to conduct a mediated moderator model in order to ascertain the mediating role of frustration.

## RESULTS

### Descriptive analyses and correlation coefficients

The means (*M*) and standard deviations (*SD*) of junior high school students' aggressive behaviors, parent**–**child affinity, parent**–**child conflict, and frustration, and the correlation coefficients among these variables are shown in Table 1.

**TABLE 1:** Descriptive analyses and correlation coefficients of the study variables.

Variables	1	2	3	4	5	*M*	*SD*
1. Grade	1	0.07	−0.10*	0.15***	0.02	—	—
2. Aggressive behavior	0.01	1	−0.34***	0.42***	0.27***	2.53	0.60
3. Parent**–**child affinity	−0.03	−0.28***	1	−0.54***	−0.12*	3.89	0.81
4. Parent**–**child conflict	0.17***	0.38***	−0.54***	1	0.12*	2.43	0.84
5. Frustration	0.03	0.31***	−0.02	0.21***	1	3.21	0.71
*M*	—	2.45	3.92	2.18	3.02	—	—
*SD*	—	0.65	0.86	0.79	0.78	—	—

*Note*: Male data are in the bottom left part; female data are in the top right part.

* *p* < .05;

*** *p* < .001.

In both boys and girls, parent**–**child affinity and parent**–**child conflict were significantly negatively correlated. Aggressive behaviors had a significantly negative correlation with parent**–**child affinity, but were positively correlated with parent**–**child conflict and frustration. In boys, frustration was significantly negatively correlated with parent**–**child conflict but had no correlation with parent**–**child affinity. In girls, frustration had a significantly positive correlation with parent**–**child conflict as well as with parent**–**child affinity.

Independent sample *t* tests were conducted with both boys and girls to investigate whether there were gender differences in the means of all variables. Results showed that the gender difference in junior high school students' aggression and parent**–**child affinity was not significant (*t* = −1.88, *p* > .05, 95% confidence interval [CI] = [−0.16, 0.003]; *t* = −0.46, *p* > .05, 95% CI = [−0.14, 0.08]). The gender difference in parent**–**child conflict was significant (*t* = 4.56, *p* < .001, 95% CI = [0.14, 0.36]), while the gender difference in frustration was also significant (*t* = 3.82, *p* < .001，95% CI = [0.29, 0.94]). It was shown that girls' parent**–**child conflict and frustration were higher than those of boys.

### The effects of polygenes on junior high school students' aggressive behaviors

SHESIS was used to analyze the Hardy–Weinberg equilibrium for each gene. The observations of the genotypes of 5‐HTTLPR, DRD2, and COMT align with the expected values, which is consistent with the Hardy–Weinberg equilibrium (*χ* ^*2*^ = 0.04, 1.33, 0.42; *p* > .05); because MAOA is distributed in the X chromosome, only girls' Hardy–Weinberg equilibrium was analyzed (*χ* ^*2*^ = 1.15, *p* > .05).

In order to avoid result deviations, prior to analyzing the research data, this study first examined dominant, recessive, and additive coding, which can further ascertain if different alleles will affect polygenetic accumulative scores (the examination of genetic coding is presented as Supplementary Materials). This process also aimed to ascertain which coding was more appropriate in this study and whether the method of polygenetic accumulation was applicable. Results showed that four genetic polymorphisms (5‐HTTLPR, MAOA‐uVNTR, COMT [rs4680], *Taq*1 of DRD2 [rs1800497]) were accumulative, and the interactions among genes were not significant. Therefore, four genetic polymorphisms were accumulated as candidate polygenic scores (CPGSs; see Supplementary Materials). It was found that CPGS predicted aggressive behavior in both boys (*R* ^2^ = 0.034, *β* = 0.38, *p* < .001) and girls (*R* ^2^ = 0.011, *β* = 0.19, *p* < .03).

### The effects of polygenic scores and parent–child relationships on junior high school students' aggressive behaviors

The correlations between CPGS and junior high school students' aggressive behaviors, parent**–**child affinity, and parent**–**child conflict are listed in Table 2. In both male and female participants, polygenic accumulative scores had a significantly positive correlation with junior high school students' aggressive behaviors and frustration, but there was no correlation with parent**–**child affinity and parent**–**child conflict. A correlation between genes and environment was ruled out.

**TABLE 2:** Correlation coefficients between candidate polygenic scores (CPGS) and other study variables.

		Grade	Aggressive behavior	Parent–child affinity	Parent–child conflict	Frustration
CPGS	Boys	−0.02	0.18**	−0.07	0.09	0.19**
	Girls	−0.04	0.10*	−0.06	0.06	0.11*

* *p* < .05;

** *p* < .01.

The effect of the interactions between parent**–**child affinity and CPGS was not significant in either male or female participants' aggressive behaviors. The effect of the interaction between parent**–**child conflict and CPGS on male participants' aggressive behaviors was significant, but it was not significant among female participants (see Table 3).

**TABLE 3:** Effects of the interactions between parent**–**child affinity and candidate polygenic scores (CPGS) on aggressive behaviors.

Models	*ΔR* ^2^	*F*	*β*	*T*	*p*	95% confidence interval	
						Upper bound	Lower bound
Model 1a							
CPGS	0.10	25.18***	0.35	3.66	<.001	0.16	0.54
Parent**–**child affinity			−0.20	−5.70	<.001	−0.27	−0.13
CPGS × Parent**–**child affinity	0.002	0.75	−0.09	−0.87	.39	−0.30	0.12
Model 2a							
CPGS	0.17	44.20***	0.31	3.40	.001	0.13	0.19
Parent**–**child conflict			0.30	8.23	<.001	0.23	0.37
CPGS × parent**–**child conflict	0.01	4.79*	0.25	2.20	.02	0.03	0.47
Model 1b							
CPGS	0.12	31.81*	0.14	1.79	.07	−0.01	0.30
Parent**–**child affinity			−0.25	−7.71	<.001	−0.32	−0.19
CPGS × Parent**–**child affinity	0.01	2.44	0.16	1.56	.12	−0.04	0.36
Model 2b							
CPGS	0.15	40.37**	0.14	1.75	.08	−0.02	0.29
Parent**–**child conflict			0.26	8.63	<.001	0.20	0.32
CPGS × parent**–**child conflict	0.002	0.95	0.09	0.98	.33	−0.09	0.26

*Note*: Model 1a and Model 2a were for boys; Model 2a and Model 2b were for girls.

* *p* < .05;

** *p* < .01;

*** *p* < .001.

**FIGURE 1:**
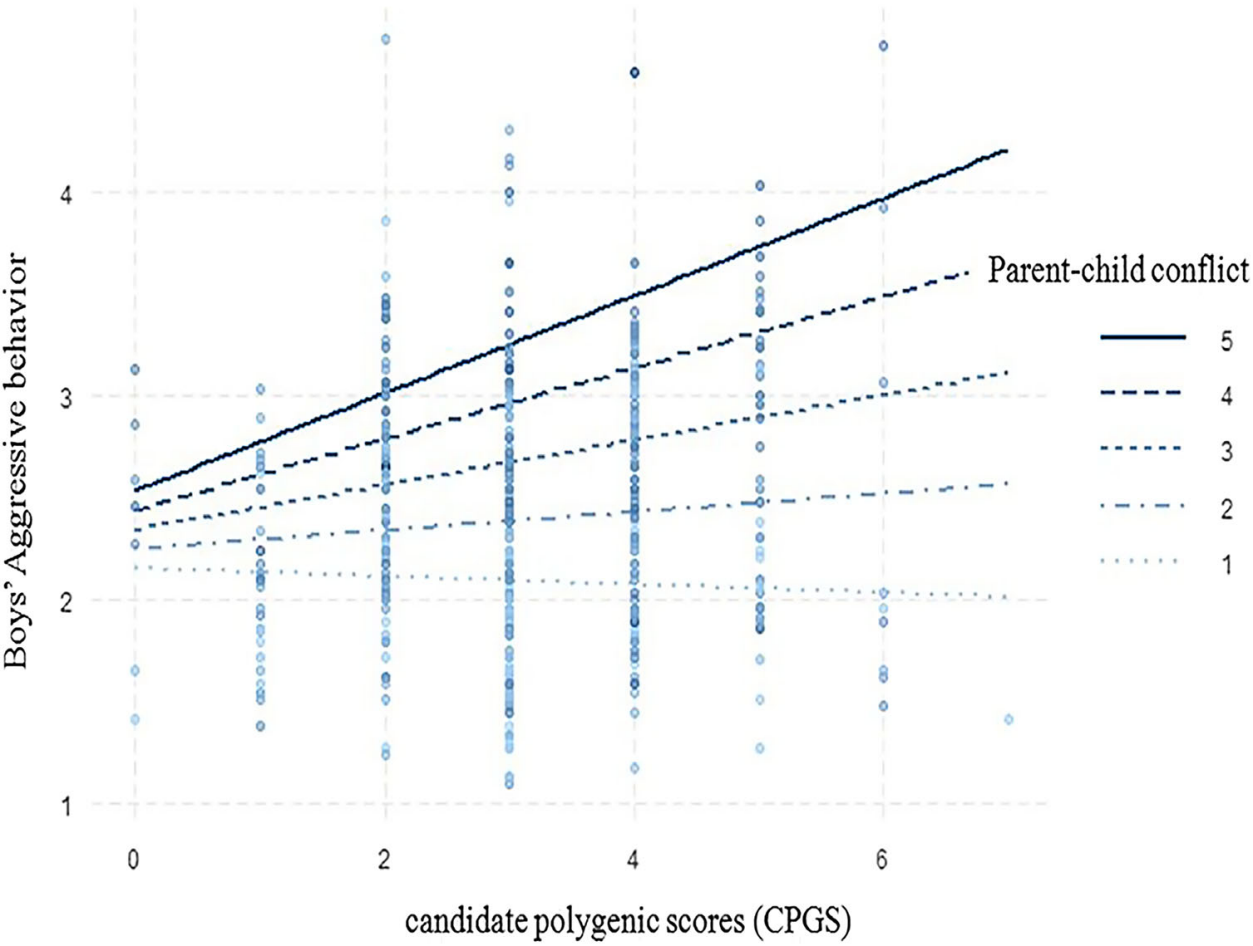
Effects of the interaction between candidate polygenic scores (CGPS) and parent–child conflict on boys' aggressive behaviors. Under the condition of high parent–child conflict (when the parent–child conflict value is 3, 4 or 5), the effect of CPGS on the aggressive behavior of male junior high school students increased; under the condition of low parent–child conflict (when the value of parent–child conflict is 1 or 2), the influence of CPGS on the aggressive behavior of male junior high school students is not significant.

A simple slope analysis was conducted to examine the effects of the interaction between parent**–**child conflict and CGPS on male participants' aggressive behaviors (see Figure 1). When parent**–**child conflict was high, the effect of CPGS on males' aggressive behaviors increased with the number of risk genes; when parent**–**child conflict was low, the effect of CPGS on male junior high school students did not change appreciably. This finding suggests that the effect of the interaction between CPGS and parent**–**child conflict on males' aggressive behaviors is consistent with the diathesis‐stress model.

### The effects of polygenic scores and parent–child relationships on junior high school students' aggressive behaviors: The role of frustration

Frustration had a significant positive prediction on junior high school students' aggressive behaviors (*p* < .001); the 95% CI of each regression coefficient did not include 0 (Table 4).

**TABLE 4:** Effect of frustration on students' aggressive behaviors.

Independent variable	Gender	*R* ^2^	*β*	*p*	95% confidence interval	
					Upper bound	Lower bound
Frustration	Boys	0.10	0.26***	<.001	0.19	0.33
	Girls	0.07	0.23***	<.001	0.15	0.30

*** *p* < .001.

**FIGURE 2:**
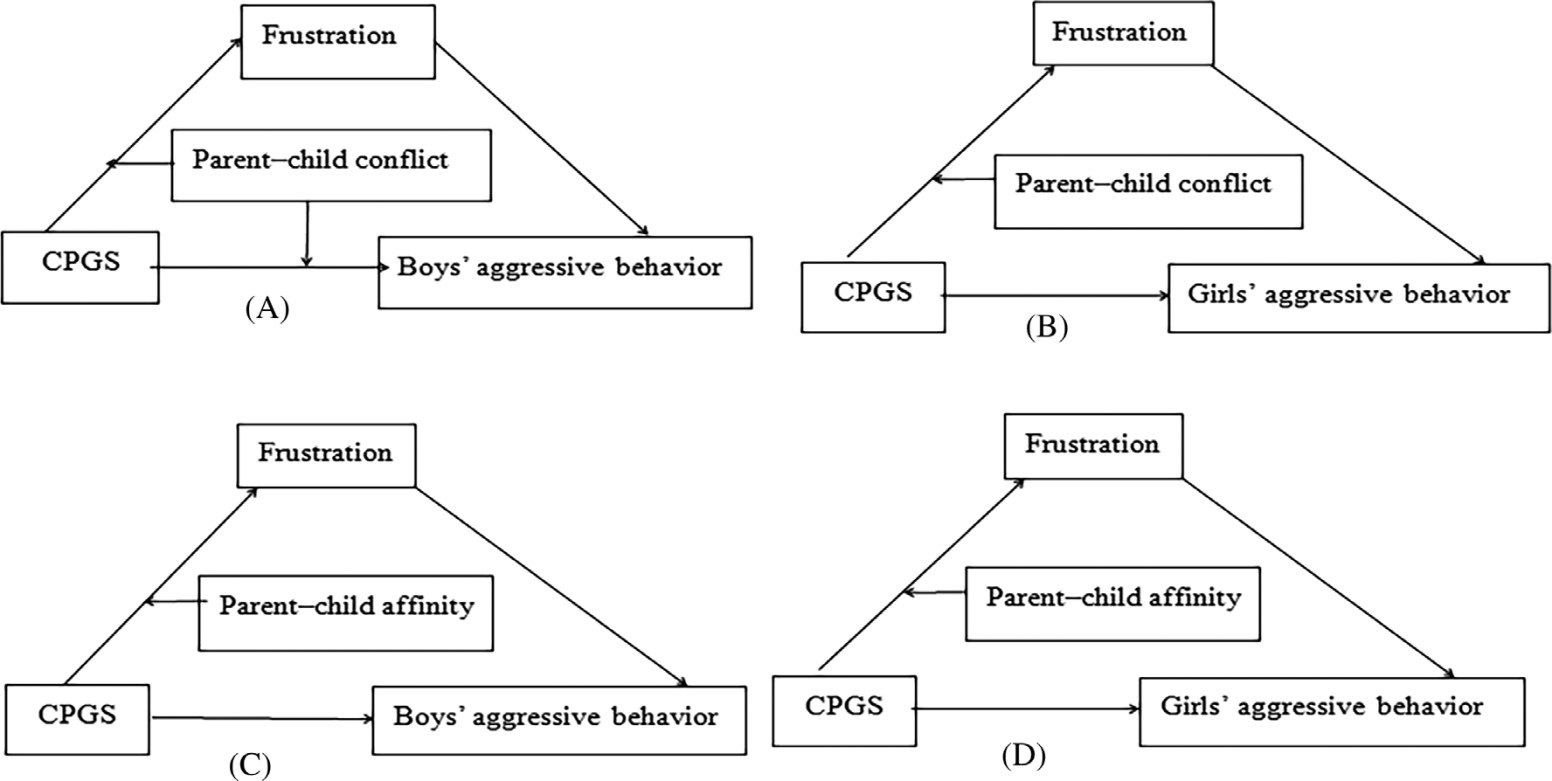
Mediation models with moderator. The interaction between parent–child conflict and polygenic cumulative scores is significant only for boys' aggressive the study constructs model (A) for boys and model (B) for girls. The interaction effects of parent–child affinity and polygenic cumulative scores on the aggressive behavior of male and female junior high school students were not significant, so models (C) and (D) were constructed.

Based on the above findings, four mediated moderator models were built (see Figure 2) to examine if the interactions between polygenic accumulative scores and parent**–**child relationship predicted junior high school students' aggressive behaviors through frustration.

**FIGURE 3:**
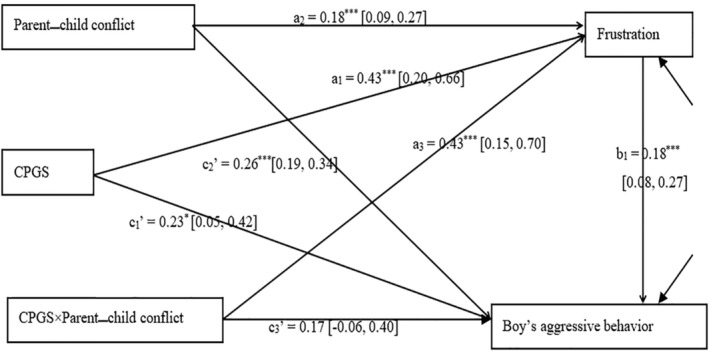
Effect of candidate polygenic scores (CPGS) on boys' aggressive behavior, with frustration as a mediator and parent–child conflict as a moderator. Values in parentheses are the 95% confidence intervals of each estimated path coefficient, as in Figure 4.

Model (A) was a fully saturated model (see Figure 3) and showed that the main effect of CPGS on frustration was significant (*β* = 0.43, *p* < .001); the main effect of parent**–**child conflict on frustration was also significant (*β* = 0.18, *p* < .001). Furthermore, the interaction between CPGS and parent**–**child conflict also significantly affected frustration (*β* = 0.43, *p* = .002). The main effect of frustration and parent**–**child conflict on male junior high school students' aggressive behaviors was significant (*β* = 0.18, *p* < .001 and *β* = 0.26, *p* < .001, respectively). The main effect of CPGS on aggressive behaviors was significant (*β* = 0.23, *p* = .01), and the interaction between CPGS and parent**–**child conflict did not significantly affect aggressive behaviors (*β* = 0.17, *p* = .11). Therefore, the path coefficients of *a*_3_ and *b*_1_ were all significant, and the mediated moderator model (A) was established. In addition, coefficient *c*_3_' was not significant, meaning that the direct mediating effect of parent**–**child conflict on the relationship between CPGS and male junior high school students' aggressive behaviors was not significant. In other words, there was a full mediator in the moderation of parent**–**child conflict. The estimated value of *a*_3_ *b*_1_ was 0.08, and its 95% CI = [0.02, 0.15]. Because this range does not include 0, *a*_3_ *b*_1_ is significant.

**FIGURE 4:**
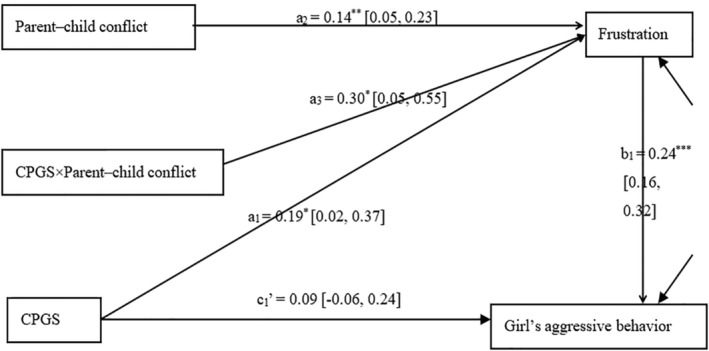
Effect of candidate polygenic scores (CPGS) on girls' aggressive behavior, with frustration as a mediator and parent–child conflict as a moderator.

The fitted values of model (B) were *χ* ^*2*^/*df* = 1.71; Tucker‐Lewis index (TLI) = 0.96; comparative fit index (CFI) = 0.99; root‐mean‐square error of approximation (RMESA) = 0.04 (see Figure 4). This model is acceptable based on fit indexes. The main effect of CPGS on female junior high school students' aggressive behaviors was not significant (*β* = 0.09, *p* = .22), but the main effect of frustration on their aggressive behaviors was significant (*β* = 0.24, *p* < .001). The main effect of CPGS on frustration was significant (*β* = 0.19, *p* = .03), and parent**–**child conflict also had a significant effect on frustration (*β* = 0.14, *p* = .003). The interaction between CPGS and parent**–**child conflict also had a significant effect on frustration (*β* = 0.30, *p* = .02). Hence, paths *a*_3_ and *b*_1_ were both significant. A bootstrap test was conducted, and it was found that the 95% CI of *a*_3_ *b*_1_ was [0.02, 0.15] (0 is not included in this range). This model was acceptable and showed that the interaction between CPGS and parent**–**child conflict affected female junior high school students' aggressive behaviors totally through frustration.

The fitting values of model (C) showed that this model was not acceptable, *χ* ^*2*^/*df* = 4.19; TLI = 0.49; CFI = 0.86; RMESA = 0.12, and the fitting values of model (D) showed that this model can be acceptable, *χ* ^*2*^/*df* = 1.83; TLI = 0.95; CFI = 0.99; RMESA = 0.04. However, the main effect of CPGS on female junior high school students' aggressive behaviors was not significant (*β* = 0.09, *p* = .24), and the interaction between CPGS and parent**–**child affinity did not have a significant effect on frustration (*β* = −0.05, *p* = .70).

## DISCUSSION

In the present study, it was found that the interaction effect of CPGS and parent**–**child relationship on junior high school students' aggressive behaviors was more consistent with the diathesis‐stress hypothesis than with the two other hypotheses, and completely mediated by frustration among boys. However, the interaction effect of CPGS and parent**–**child affinity on aggression was not affected by frustration. These results are further reflected upon in the following sections so as to better understand their contributions to the extant literature.

### The polygenic hereditary basis of junior high school students' aggressive behaviors

The candidate gene method adopted in this study explored the joint effects of four genes on junior high school students' aggressive behaviors. The results showed that the four genes affect junior high school students' aggressive behaviors in an accumulative manner. Previous studies have also supported the idea that the accumulative pattern of genes affects aggressive behaviors (Nikolova et al., 2011). This effect is more salient among males. Aggressive behaviors are more likely to be hereditary; the explanation rate of polygenic accumulation towards aggressive behaviors is 3.4% in males, but only 1.1% in females. This finding also coincides with the results of the meta‐analysis conducted by Craig and Halton (2009). Furthermore, genes in this study relate mainly to the prefrontal region and amygdala, which further confirms the important roles of the prefrontal region, amygdala, and amygdala–prefrontal circuitry in the regulation of aggressive behaviors. The irregular activities of serotonin and dopamine may result in inadequate regulation from the prefrontal region and increased amygdala reactions, further leading to potential aggressive behaviors (Rosell & Siever, 2015; Siever, 2008).

### The effects of the interactions between polygenic scores and parent–child relationships on junior high school students' aggressive behaviors

The results from this study indicate that the effect of the interaction between polygenic accumulative scores and parent**–**child conflict on male junior high school students' aggressive behaviors could be explained best by the diathesis‐stress model. This result is also consistent with previous studies on the effects of the interactions between a single gene and environment on behavior (e.g., Chhangur et al., 2015). However, Fine et al. (2016) found that the effects of the interactions between genes and environment on adolescents' delinquent behaviors were better explained by the differential susceptibility model. Although this study also focused on monoamine neurotransmitters (DRD4, DRD2, DAT1), the environmental factor was teacher**–**student relationships. In contrast, the present study focused on parent**–**child relationships. The difference between the two studies indicates that the effects of the interaction between genes and environment on behaviors should not only be based on the positive and negative side of a single environmental factor, but rather that multiple environment factors should be considered.

In this study, the effects of the interactions between polygenic accumulative scores and parent**–**child conflict were found only in boys: more parent**–**child conflicts led to increased effects of CPGS on male junior high school students' aggressive behaviors. However, this finding cannot be applied to female junior high school students. Extant studies have focused mainly on understanding how genes affect problematic behaviors in a negative environment (Zhang et al., 2017), but the effects of genes on behaviors in a positive environment have not been widely explored. Therefore, this study focused on parent**–**child affinity, a positive environmental factor, but it did not find effects of the interactions between parent**–**child affinity and polygenic accumulative scores on junior high school students' aggressive behaviors. However, this finding still does not prove that the prediction of polygenic accumulation on females' aggressive behaviors is not affected by the parent**–**child relationship. The integrative theoretical model put forward by van Goozen et al. (2007) showed how the interplay between environmental factors and genes affects anti‐social behaviors through emotions. Based on this model, it is likely that the interplay between polygenes and the parent**–**child relationship can affect junior high school students' aggressive behaviors via emotions and temperaments.

### The effects of polygenes and parent–child relationships on junior high school students' aggressive behaviors: The role of frustration

This study showed that the interactions between polygenic accumulative scores and patent–child conflict affect junior high school students' aggressive behaviors and were completely mediated by frustration. This finding partially supports the theoretical framework put forward by van Goozen et al. (2007), and shows how the effects of the interactions between family environment and genes on anti‐social behaviors (e.g., aggressive behaviors) are mediated by emotion‐related endophenotypes. It also expands the gene‐endophenotype‐behavior framework to polygenes × environment‐endophenotype‐behavior. In addition, the direct effect of polygenic accumulative scores on male aggressive behaviors was significant, but the effect was not significant in females. More specifically, some parts of the hereditary basis of males' aggressive behaviors stem from the effects of polygenes, while other parts are from the joint effects of polygenes, parent**–**child conflict, and frustration. The hereditary basis of females' aggressive behaviors is wholly the result of the joint effects of polygenes, parent**–**child conflict, and frustration. This finding shows that the aggressive behaviors of males are influenced by heredity to a greater extent than those of females. However, there is still a dearth of research on females' aggressive behaviors from a neurobiological perspective, which imposes a barrier to accurately explaining gender differences in the hereditary basis of aggressive behaviors (Been et al., 2019).

This study did not find that the interactions between polygenic accumulative scores and parent**–**child affinity affected junior high school students' aggressive behaviors. A possible explanation might be that the interactions between polygenes and positive parent**–**child relationships are related only to positive individual characteristics. For instance, Belsky and Beaver (2011) found that the interactions between polygenic accumulative scores and positive parenting styles predicted higher self‐regulation, while negative parenting styles predicted lower self‐regulation. Frustration and aggressive behaviors in this study are negative individual characteristics; thus, there were no significant effects of the interactions between polygenic accumulation and parent**–**child affinity on these two variables.

### Limitations

There are some limitations in this study. First, this study selected four loci related to serotonin and dopamine in monoamine neurotransmitters. The findings from this study demonstrate that these four genic loci affect aggressive behaviors in an accumulative manner. However, the oxytocin receptor gene and the glucocorticoid receptor gene have also been found to be related to aggressive behaviors (Li et al., 2017; Malik et al., 2012). These oxytocin‐ and cortisol‐related genes may have different interactions with monoamine neurotransmitter genes. Therefore, future studies could include genes such as the oxytocin receptor gene and the glucocorticoid receptor gene in order to better understand the neurobiological mechanism of aggressive behaviors. Second, according to the theoretical framework put forward by van Goozen et al. (2007), various factors might affect the effects of the interactions between genes and environment on aggressive behaviors. Some studies found that the structure and function of the amygdala, prefrontal cortex, anterior cingulate cortex, striatum, and hypothalamus were affected by systemic genes such as serotonin and dopamine (e.g., Nikolova et al., 2011; Waller et al., 2016). These genes determine the activity level of neurotransmitters and hormones such as serotonin, dopamine, and oxytocin (Carter, 2014; Ullsperger, 2010), while the production and release of neurotransmitters and hormones are also controlled by the brain. Therefore, it can be seen that genes, the brain, and transmitters are closely interrelated. Future studies could continue to explore the role of brain function and neurotransmitters in the interactions between polygenes and aggressive behaviors.

## CONFLICT OF INTEREST STATEMENT

The authors declare there are no conflicts of interest.

## ETHICS STATEMENT

This study obtained ethics approval from the Ethical Review Board of Ludong University. Written informed consents were obtained from the adolescence and their parents.

## Supporting information


**Supplementary material S1.** The examination of genetic coding.

## References

[pchj717-bib-0001] Aliev, F. , Latendresse, S. J. , Bacanu, S. A. , Neale, M. C. , & Dick, D. M. (2014). Testing for measured gene‐environment interaction: Problems with the use of cross‐product terms and a regression model reparameterization solution. Behavior Genetics, 44(2), 165–181. 10.1007/s10519-014-9642-1 24531874 PMC4004105

[pchj717-bib-0002] Anderson, C. A. , & Bushman, B. J. (2002). Human aggression. Annual Review of Psychology, 53, 27–51. 10.1146/annurev.psych.53.100901.135231 11752478

[pchj717-bib-0003] Armstrong‐Carter, E. , Wertz, J. , & Domingue, B. W. (2021). Genetics and child development: Recent advances and their implications for developmental research. Child Development Perspectives, 15(1), 57–64. 10.1111/cdep.12400

[pchj717-bib-0004] Åslund, C. , Comasco, E. , Nordquist, N. , Leppert, J. , Oreland, L. , & Nilsson, K. W. (2013). Self‐reported family socioeconomic status, the 5‐HTTLPR genotype, and delinquent behavior in a community‐based adolescent population. Aggressive Behavior, 39(1), 52–63. 10.1002/ab.21451 22987641

[pchj717-bib-0005] Beaver, K. M. , Wright, J. P. , De Lisi, M. , Walsh, A. , Vaughn, M. G. , Boisvert, D. , et al. (2007). A gene × gene interaction between DRD2 and DRD4 is associated with conduct disorder and antisocial behavior in males. Behavioral and Brain Functions, 3(1), 30. 10.1186/1744-9081-3-30 17587443 PMC1913922

[pchj717-bib-0006] Been, L. E. , Gibbons, A. B. , & Meisel, R. L. (2019). Towards a neurobiology of female aggression. Neuropharmacology, 156, 107451. 10.1016/j.neuropharm.2018.11.039 30502376 PMC6939635

[pchj717-bib-0007] Belsky, J. , & Beaver, K. M. (2011). Cumulative‐genetic plasticity, parenting and adolescent self‐regulation. Journal of Child Psychology and Psychiatry, 52(5), 619–626. 10.1111/j.1469-7610.2010.02327.x 21039487 PMC4357655

[pchj717-bib-0008] Belsky, J. , Jonassaint, C. , Pluess, M. , Stanton, M. , Brummett, B. , & Williams, R. (2009). Vulnerability genes or plasticity genes? Molecular Psychiatry, 14(8), 746–754. 10.1038/mp.2009.44 19455150 PMC2834322

[pchj717-bib-8002] Bouchard , & Thomas, J. (2004). Genetic influence on human psychological traits. A survey. Current Directions in Psychological Science, 13(4), 148–151.

[pchj717-bib-0010] Buss, A. H. , & Perry, M. (1992). The aggression questionnaire. Journal of Personality and Social Psychology, 63(3), 452–459. 10.1037//0022-3514.63.3.452 1403624

[pchj717-bib-0011] Byrd, A. L. , & Manuck, S. B. (2014). MAOA, childhood maltreatment, and antisocial behavior: Meta‐analysis of a gene‐environment interaction. Biological Psychiatry, 75(1), 9–17. 10.1016/j.biopsych.2013.05.004 23786983 PMC3858396

[pchj717-bib-0013] Carter, C. S. (2014). Oxytocin pathways and the evolution of human behavior. Annual Review of Psychology, 65, 17–39. 10.1146/annurev-psych-010213-115110 24050183

[pchj717-bib-0014] Caspi, A. , McClay, J. , Moffitt, T. E. , Mill, J. , Martin, J. , Craig, I. W. , Taylor, A. , & Poulton, R. (2002). Role of genotype in the cycle of violence in maltreated children. Science, 297(5582), 851–854. 10.1126/science.1072290 12161658

[pchj717-bib-0015] Chan, R. C. K. , Yang, B. , & Wang, Y. (2008). Application of endophenotype approach in psychiatric research. Psychological Science, 16(3), 378–391. http://journal.psych.ac.cn/xlkxjz/EN/Y2008/V16/I03/378

[pchj717-bib-0016] Chhangur, R. R. , Overbeek, G. , Verhagen, M. , Weeland, J. , Matthys, W. , & Engels, R. C. M. E. (2015). DRD4 and DRD2 genes, parenting, and adolescent delinquency: Longitudinal evidence for a gene by environment interaction. Journal of Abnormal Psychology, 124(4), 791–802. 10.1037/abn0000091 26595468

[pchj717-bib-0017] Cleveland, H. H. , Schlomer, G. L. , Vandenbergh, D. J. , Feinberg, M. , Greenberg, M. , Spoth, R. , Redmond, C. , Shriver, M. D. , Zaidi, A. A. , & Hair, K. L. (2015). The conditioning of intervention effects on early adolescent alcohol use by maternal involvement and dopamine receptor D4 (DRD4) and serotonin transporter linked polymorphic region (5‐HTTLPR) genetic variants. Development and Psychopathology, 27(1), 51–67. 10.1017/S0954579414001291 25640830 PMC4450765

[pchj717-bib-0018] Craig, I. W. , & Halton, K. E. (2009). Genetics of human aggressive behaviour. Human Genetics, 126, 101–113. 10.1007/s00439-009-0695-9 19506905

[pchj717-bib-0019] Davies, P. T. , & Cicchetti, D. (2014). How and why does the 5‐HTTLPR gene moderate associations between maternal unresponsiveness and children's disruptive problems? Child Development, 85(2), 484–500. 10.1111/cdev.12148 24033129 PMC4557734

[pchj717-bib-0020] De Laet, S. , Colpin, H. , van Leeuwen, K. , Wim, V. D. N. , Claes, S. , Janssens, A. , Goossens, L. , & Verschueren, K. (2016). Teacher‐student relationships and adolescent behavioral engagement and rule‐breaking behavior: The moderating role of dopaminergic genes. Journal of School Psychology, 56, 13–25. 10.1016/j.jsp.2016.02.002 27268567

[pchj717-bib-0021] Ellis, L. K. , & Rothbart, M. K. (2001). Revision of the early adolescent temperament questionnaire. Poster presented at the Biennial Meeting of the Society for Research in Child Development in Minneapolis, Minnesota.

[pchj717-bib-0022] Fernàndez‐Castillo, N. , & Cormand, B. (2016). Aggressive behavior in humans: Genes and pathways identified through association studies. American Journal of Medical Genetics Part B: Neuropsychiatric Genetics, 171(5), 676–696. 10.1002/ajmg.b.32419 26773414

[pchj717-bib-8004] Fine, A. , Mahler, A. , Simmons, C. , Chen, C. , Moyzis, R. , & Cauffman, E. (2016). Relations between three dopaminergic system genes, school attachment, and adolescent delinquency. Developmental Psychology, 52(11), 1893–1903. 27786532 10.1037/dev0000166

[pchj717-bib-0023] Furman, W. , & Buhrmester, D. (1985). Children's perceptions of the personal relationships in their social networks. Developmental Psychology, 21(6), 1016–1024. 10.1037/0012-1649.21.6.1016

[pchj717-bib-0024] Harden, K. P. , & Koellinger, P. D. (2020). Using genetics for social science. Nature Human Behaviour, 4, 567–576. 10.1038/s41562-020-0862-5 PMC8240138 32393836

[pchj717-bib-8005] IBM Corp . (2013). SPSS Statistic for Windows (Version 22.0). IBM Corp.

[pchj717-bib-0025] Jolicoeur‐Martineau, A. , Belsky, J. , Szekely, E. , Widaman, K. , Pluess, M. , Greenwood, C. , & Wazana, A. (2017). Distinguishing differential susceptibility, diathesis‐stress, and vantage sensitivity: Beyond the single gene and environment model. Development and Psychopathology, 32(1), 73–83. 10.1017/S0954579418001438 30626458

[pchj717-bib-0026] Kim‐Cohen, J. , Caspi, A. , Taylor, A. , Williams, B. , Newcombe, R. , Craig, I. W. , & Moffitt, T. E. (2006). MAOA, maltreatment, and gene–environment interaction predicting children's mental health: New evidence and a meta‐analysis. Molecular Psychiatry, 11, 903–913. 10.1038/sj.mp.4001851 16801953

[pchj717-bib-0027] Langevin, S. , Mascheretti, S. , Côté, S. M. , Vitaro, F. , Boivin, M. , Turecki, G. , Tremblay, R. E. , & Ouellet‐Morin, I. (2019). Cumulative risk and protection effect of serotonergic genes on male antisocial behaviour: Results from a prospective cohort assessed in adolescence and early adulthood. The British Journal of Psychiatry, 214(3), 137–145. 10.1192/bjp.2018.251 30774060

[pchj717-bib-0028] Li, J. , Tang, L. , Wang, Y. , Li, F. , Bao, M. , Xiang, J. , Lei, D. , & Tang, B. (2017). Genetic associations and interactions between the NR3C1 (GR) and NR3C2 (MR) genes and aggressive behavior in a central south Chinese Han population. Genetic Testing and Molecular Biomarkers, 21(8), 497–505. 10.1089/gtmb.2016.0417 28686058

[pchj717-bib-0029] Malik, A. I. , Zai, C. C. , Abu, Z. , Nowrouzi, B. , & Beitchman, J. H. (2012). The role of oxytocin and oxytocin receptor gene variants in childhood‐onset aggression. Genes, Brain, and Behavior, 11(5), 545–551. 10.1111/j.1601-183X.2012.00776.x 22372486

[pchj717-bib-8006] Muthén, L. K. , & Muthén, B. O. (1998‐2017). Mplus user's guide (7th ed.). Muthén & Muthén.

[pchj717-bib-0030] Nikolova, Y. S. , Ferrell, R. E. , Manuck, S. B. , & Hariri, A. R. (2011). Multilocus genetic profile for dopamine signaling predicts ventral striatum reactivity. Neuropsychopharmacology, 36, 1940–1947. 10.1038/npp.2011.82 21593733 PMC3154113

[pchj717-bib-0033] Pawliczek, C. M. , Derntl, B. , Kellermann, T. , Gur, R. C. , Schneider, F. , & Habel, U. (2013). Anger under control: Neural correlates of frustration as a function of trait aggression. PLoS One, 8(10), e78503. 10.1371/journal.pone.0078503 24205247 PMC3799631

[pchj717-bib-0034] Pluess, M. (2017). Vantage sensitivity: Environmental sensitivity to positive experiences as a function of genetic differences. Journal of Personality, 85(1), 38–50. 10.1111/jopy.12218 26271007

[pchj717-bib-0035] Pluess, M. , & Belsky, J. (2013). Vantage sensitivity: Individual differences in response to positive experiences. Psychological Bulletin, 139(4), 901–916. 10.1037/a0030196 23025924

[pchj717-bib-0036] Raffington, L. , Mallard, T. , & Harden, K. P. (2020). Polygenic scores in developmental psychology: Invite genetics in, leave biodeterminism behind. Annual Review of Developmental Psychology, 2(1), 389–411. 10.1146/annurevdevpsych-051820-123945 PMC10798791 38249435

[pchj717-bib-0037] Reif, A. , Rösler, M. , Freitag, C. M. , Schneider, M. , Eujen, A. , Kissling, C. , Wenzler, D. , Jacob, C. P. , Retz‐Junginger, P. , Thome, J. , Lesch, K. , & Retz, W. (2007). Nature and nurture predispose to violent behavior: Serotonergic genes and adverse childhood environment. Neuropsychopharmacology, 32, 2375–2383. 10.1038/sj.npp.1301359 17342170

[pchj717-bib-0038] Rohner, R. P. , & Britner, P. A. (2002). Worldwide mental health correlates of parental acceptance‐rejection: Review of cross‐cultural and intracultural evidence. Cross‐Cultural Research, 36(1), 16–47. 10.1177/106939710203600102

[pchj717-bib-0039] Rosell, D. R. , & Siever, L. J. (2015). The neurobiology of aggression and violence. CNS Spectrums, 20(3), 254–279. 10.1017/S109285291500019X 25936249

[pchj717-bib-0040] Sentse, M. , & Laird, R. D. (2010). Parent–child relationships and dyadic friendship experiences as predictors of behavior problems in early adolescence. Journal of Clinical Child & Adolescent Psychology, 39(6), 873–884. 10.1080/15374416.2010.517160 21058133

[pchj717-bib-8003] Shi, Y. Y. , & He, L. (2005). SHEsis, a powerful software platform for analyses of linkage disequilibrium, haplotype construction, and genetic association at polymorphism loci. Cell Research, 15(2), 97–98. 10.1038/sj.cr.7290272 15740637

[pchj717-bib-0041] Siever, L. J. (2008). Neurobiology of aggression and violence. American Journal of Psychiatry, 165(4), 429–442. 10.1176/appi.ajp.2008.07111774 18346997 PMC4176893

[pchj717-bib-0043] Tielbeek, J. J. , Johansson, A. , Polderman, T. J. , Rautiainen, M. R. , Jansen, P. , Taylor, M. , Tong, X. , Lu, Q. , Burt, A. S. , Tiemeier, H. , Viding, E. , Plomin, R. , Martin, N. G. , Heath, A. C. , Madden, P. A. F. , Montgomery, G. , Beaver, K. M. , Waldman, I. , Gelernter, J. , … Posthuma, D. (2017). Genome‐wide association studies of a broad spectrum of antisocial behavior. JAMA Psychiatry, 74(12), 1242–1250. 10.1001/jamapsychiatry.2017.3069 28979981 PMC6309228

[pchj717-bib-8001] Tielbeek, J. J. , Medland, S. E. , Benyamin, B. , Byrne, E. M. , Heath, A. C. , Madden, P. A. , Martin, N. G. , Wray, N. R. , Verweij, K. J. H. (2012). Unraveling the genetic etiology of adult antisocial behavior: A genome‐wide association study. PLoS One, 7(10), e45086. 10.1371/journal.pone.0045086 PMC3471931 23077488

[pchj717-bib-0044] Tuvblad, C. , & Baker, L. A. (2011). Human aggression across the lifespan: Genetic propensities and environmental moderators. Advances in Genetics, 75, 171–214. 10.1016/B978-0-12-380858-5.00007-1 22078481 PMC3696520

[pchj717-bib-0045] Uchiyama, R. , Spicer, R. , & Muthukrishna, M. (2021). Cultural evolution of genetic heritability. Behavioral and Brain Sciences, 45, e152. 10.1017/S0140525X21000893 34016199

[pchj717-bib-0046] Ullsperger, M. (2010). Genetic association studies of performance monitoring and learning from feedback: The role of dopamine and serotonin. Neuroscience and Biobehavioral Reviews, 34(5), 649–659. 10.1016/j.neubiorev.2009.06.009 19563825

[pchj717-bib-0047] van Goozen, S. H. , Fairchild, G. , Snoek, H. , & Harold, G. T. (2007). The evidence for a neurobiological model of childhood antisocial behavior. Psychological Bulletin, 133(1), 149–182. 10.1037/0033-2909.133.1.149 17201574

[pchj717-bib-0048] Waller, R. , Corral‐Frias, N. S. , Vannucci, B. , Bogdan, R. , Knodt, A. R. , Hariri, A. R. , & Hyde, L. W. (2016). An oxytocin receptor polymorphism predicts amygdala reactivity and antisocial behavior in men. Social Cognitive and Affective Neuroscience, 11(8), 1218–1226. 10.1093/scan/nsw042 27036876 PMC4967804

[pchj717-bib-0051] Zhang, Y. , Ming, Q. S. , Yi, J. Y. , Wang, X. , Chai, Q. L. , & Yao, S. Q. (2017). Gene‐gene‐environment interactions of serotonin transporter, monoamine oxidase a and childhood maltreatment predict aggressive behavior in Chinese adolescents. Frontiers in Behavioral Neuroscience, 11, 17. 10.3389/fnbeh.2017.00017 28203149 PMC5285338

[pchj717-bib-0052] Zhao, J. X. , Liu, X. , & Zhang, W. X. (2013). Peer rejection, peer acceptance and psychological adjustment of left‐behind children: The roles of parental cohesion and children's cultural beliefs about adversity. Acta Psychologica Sinica, 45(7), 797–810. 10.3724/SP.J.1041.2013.00797

