# Supplementary material for: Effects of polygenes, parent–child relationship and frustration on junior high school students' aggressive behaviors
Source: Psych J. 2023 Dec 27;13(2):265–75. doi: 10.1002/pchj.717 (PMC10990803; doi:10.1002/pchj.717)
Supplement: Supplementary file 1 — Supplementary material S1. The examination of genetic coding. [file PCHJ-13-265-s001.docx]

**The examination of genetic coding**

The genetic coding mainly considers dominant genetic coding, recessive genetic coding, and additive genetic coding. Dominant genetic coding regards risk genes as dominant expressions; as long as there is one risk gene, the code 1 will be applied. Recessive genetic coding regards risk genes as recessive expressions, and only with two risk genes can they be coded as 1. Additive genetic coding means that carrying two risk genes is coded as 2, while carrying one risk gene is coded as 1. Research has shown that there might be ethnic and gender differences in the effects of each genotype on behaviors; these studies’ results are also inconsistent (Ficks & Waldman, 2014). Therefore, this study first evaluates the risk of each genotype, and uses the genotypes of 5-HTTLPR S/L, MAOA 3R/3R (3R), COMT Val/Val, and DRD2 A1/A1 as references to conduct the dummy coding in order to examine the effects of each genotype on aggressive behaviors, and further evaluate which genotypic coding is the risk gene. Three coding schemes for each genetic polymorphism are shown in Table S1.

Table S1 Three coding schemes for 5-HTTLPR, MAOA, COMT, and DRD2 genotypes

| Gene | Additive coding | Dominant coding | Recessive coding |
| --- | --- | --- | --- |
| 5-HTTLPR | Boys L/L=2, S/L=1, S/S=0  Girls S/S=2，S/L=1，L/L=0 | Boys L/L=1, S/L=1, S/S=0  Girls S/S=1, S/L=1, L/L=0 | Boys L/L=1, S/L=0, S/S=0  Girls S/S=1, S/L=0, L/L=0 |
| MAOA | 4R/4R(4R)=2,  4R/3R=1, 3R/3R(3R)=0 | 4R/4R(4R)=1,  4R/3R=1, 3R/3R(3R)=0 | 4R/4R(4R)=1,  4R/3R=0, 3R/3R(3R)=0 |
| COMT | Val/Val=2, Val/Met=1,  Met/Met=0 | Val/Val=1, Val/Met=1,  Met/Met=0 | Val/Val=1,  Val/Met=0, Met/Met=0 |
| DRD2 | A2/A2=2, A1/A2=1, A1/A1=0 | A2/A2=1, A1/A2=1, A1/A1=0 | A2/A2=1, A1/A2=0, A1/A1=0 |

Results showed that in boys, the scores of aggressive behaviors in the 5-HTTLPR L/L genotype are high and the scores of aggressive behaviors in girls’ 5-HTTLPR S/S genotype are higher (see Table S2). Moreover, the scores of aggressive behaviors of MAOA 4R, COMT Val, and DRD2 A carriers are relatively high. Hence, in boys, the genotypes of 5-HTTLPR L/L, MAOA 4R, COMT Val/Val, and DRD2 A2/A2 are risk genes, while in girls, the genotypes of 5-HTTLPR S/S, MAOA 4R/4R, COMT Val/Val, and DRD2 A2/A2 are risk genes.

Table S2 Effects of each gene on junior school students’ aggressive behaviors

|  |  | Boys’ aggressive behaviors | | |  | Girls’ aggressive behaviors | | |
| --- | --- | --- | --- | --- | --- | --- | --- | --- |
| Gene | Genotype | ΔR2 | β | p |  | ΔR2 | β | p |
| 5-HTTLPR | L/L | 0.021 | 0.07* | 0.02 |  | 0.013 | 0.09 | 0.38 |
|  | S/S |  | -0.08 | 0.20 |  |  | 0.14* | 0.02 |
| MAOA | 4R/4R(4R) | 0.005 | 0.16* | 0.01 |  | 0.001 | 0.001 | 0.997 |
|  | 4R/3R |  |  |  |  |  | 0.02 | 0.85 |
| COMT | Met/Met | 0.007 | -0.18 | 0.11 |  | 0.001 | -0.08 | 0.46 |
|  | Val/Met |  | 0.01 | 0.84 |  |  | -0.02 | 0.73 |
| DRD2 | A2/A2 | 0.008 | 0.10 | 0.25 |  | 0.011 | 0.20* | 0.02 |
|  | A1/A2 |  | 0.13 | 0.06 |  |  | 0.04 | 0.51 |

* p<0.05, ** p<0.01, *** p<0.001.

In addition, when calculating the accumulative scores of multiple candidate genes, if there are different hereditary effects in different genetic loci, then there might be a chance of obtaining a false positive or negative result. In order to avoid result deviations, prior to analyzing research data, dominant, recessive, and additive coding were first examined, and a procedure that can help us ascertain if different alleles would affect polygenetic accumulative scores. This process also aimed to identify which coding is more appropriate in this study and whether the method of polygenetic accumulation is applicable (Cao, 2017). Results showed that four genetic polymorphisms (5-HTTLPR, MAOA-uVNTR, COMT (rs4680), DRD2 (rs1800497)) can be accumulative (Table S3), and the interactions among genes were not significant (Table S4). Therefore, four genetic polymorphisms were accumulated as candidate polygenic scores (CPGS).

Table S3 comparisons among three models

| Dependent variable | Models | Model fitting |  |  | Model Change |  |
| --- | --- | --- | --- | --- | --- | --- |
|  |  | ΔR2 | F(df) |  | ΔR2 | F(df) |
| Boys’ aggressive behavior | Decomposition model | 0.033 | 1.62(9, 426) |  |  |  |
|  | Linear gene effect model | 0.024 | 2.12(5, 430) |  | 0.009 | 0.97(4, 426) |
|  | Isogene effect model | 0.021 | 4.64(2, 433) |  | 0.003 | 0.43(3, 430) |
| Girls’ aggressive behavior | Decomposition model | 0.032 | 1.64(9, 446) |  |  |  |
|  | Linear gene effect model | 0.021 | 1.93(5, 450) |  | 0.011 | 1.24(4, 446) |
|  | Isogene effect model | 0.014 | 3.22(2, 453) |  | 0.007 | 1.06(3, 450) |

Table S4 Effects of polygenic interactions on junior high school students’ aggressive behaviors

|  | Boys’ aggressive behaviors |  | | Girls’ aggressive behaviors | |
| --- | --- | --- | --- | --- | --- |
|  | t | p |  | t | P |
| 5-HTTLPR | 2.40 | 0.02 |  | 1.77 | 0.08 |
| MAOA | 1.57 | 0.12 |  | 0.14 | 0.89 |
| COMT | 0.78 | 0.44 |  | 1.69 | 0.09 |
| DRD2 | 1.07 | 0.28 |  | 1.23 | 0.22 |
| 5-HTTLPR × MAOA | 0.16 | 0.88 |  | -0.58 | 0.56 |
| 5-HTTLPR × DRD2 | -0.74 | 0.46 |  | -0.83 | 0.41 |
| MAOA × COMT | 0.81 | 0.42 |  | -0.25 | 0.81 |
| MAOA × DRD2 | 1.79 | 0.08 |  | 0.40 | 0.69 |
| COMT × DRD2 | 0.61 | 0.54 |  | -1.29 | 0.20 |
| 5-HTTLPR × MAOA × COMT | -0.69 | 0.49 |  | 6.77 | 0.50 |
| 5-HTTLPR × MAOA × DRD2 | 0.54 | 0.59 |  | 0.47 | 0.64 |
| 5-HTTLPR × COMT × DRD2 | -0.08 | 0.93 |  | 1.01 | 0.31 |
| MAOA × COMT × DRD2 | 0.96 | 0.34 |  | -0.40 | 0.69 |
| 5-HTTLPR × MAOA × COMT × DRD2 | -1.78 | 0.08 |  | 0.51 | 0.61 |

Note: Based on the examination of genotypical coding schemes, linear regression was used to analyze if there are interactions among genes. Results showed that in neither boys nor girls, there were no significant effects of each gene in two-factor, three-factor, four-factor interactions on junior high school students’ aggressive behaviors (p > 0.05).
